# Cognitive impairment and all‐cause mortality among Chinese adults aged 80 years or older

**DOI:** 10.1002/brb3.2325

**Published:** 2021-09-07

**Authors:** Yaxi Li, Heng Jiang, Xurui Jin, Huali Wang, John S. Ji, Lijing L. Yan

**Affiliations:** ^1^ Global Health Research Center Duke Kunshan University Kunshan China; ^2^ EHESP French School of Public Health Rennes France; ^3^ Dementia Care and Research Center Peking University Institute of Mental Health Beijing China; ^4^ Environmental Research Center Duke Kunshan University Kunshan China; ^5^ Nicholas School of the Environment Duke University Durham North Carolina USA; ^6^ Duke Global Health Institute Duke University Durham North Carolina USA; ^7^ Institute for Global Health and Management Peking University Beijing China; ^8^ School of Health Sciences Wuhan University Wuhan China

**Keywords:** cognitive impairment, mortality, oldest‐old, subdomain, subgroup, time‐dependent Cox regression

## Abstract

**Objectives:**

The oldest‐old (aged ≥80 years) are the fastest growing population segment and age is related to cognitive impairment. We aimed to estimate the association between cognitive impairment and all‐cause mortality, in addition to the relationship with different cognitive subdomains among the oldest‐old in China.

**Methods:**

We analyzed 25,285 participants recruited from 22 out of 30 provinces in the Chinese Longitudinal Healthy Longevity Survey (CLHLS) from 1998 to 2008, with mortality follow‐up until 2014. Cognitive function was measured by the Chinese‐version 30‐item Mini‐Mental State Examination (MMSE), classified as no (MMSE score: 25–30), mild (18–24), moderate (10–17), and severe (0–9) impairment. We used time‐dependent Cox model to evaluate the relationship between time‐varying cognition and mortality.

**Results:**

The relationship between cognition and mortality showed a dose–response pattern among the overall population. Compared to those with no impairment, participants with moderate (HR = 1.41, 95% CI 1.28–1.56) and severe (HR = 1.77, 95% CI 1.59–1.96) cognitive impairment showed increased mortality risk. Impairment in the subdomain of orientation was independently associated with increased mortality risk (HR = 1.20, 95% CI 1.05–1.36) among participants without overall cognitive impairment. Urban and rural residents had similar mortality risk.

**Conclusions:**

A consistent dose–response pattern existed between cognitive impairment and all‐cause mortality. Orientation was associated with mortality in the population without cognitive impairment. Similar mortality regardless of residence areas indicated scarce health care and treatment for cognitive impairment in China from 1998 to 2014.

## Graphical abstract

Based on 25,285 Chinese participants 80 years or older, we found a clear negative dose–response relationship between the severity of time‐varying cognitive impairment and the probability of survival for both female and male and the total population. The relationship between cognition and mortality was similar for urban and rural residents, and the dose–response pattern disappeared in the highest educated group. The orientation subscale was associated with mortality risk independent of the overall MMSE score.

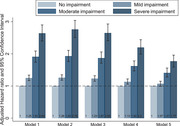

## 1 INTRODUCTION

Ageing‐related health problems are becoming increasingly important in health policies and maintaining sustainable development globally (United Nations, Department of Economic & Social Affairs, Population Division, 2017). Compared to all older adults aged 60 years or above, the number of oldest‐old (aged ≥80) is projected to rise faster, by threefold by 2050 and nearly seven times by 2100 than that in 2017 (United Nations, Department of Economic & Social Affairs, Population Division, 2017). Cognitive impairment is one of the major aging‐related conditions, characterized by impaired capacity to remember, learn, concentrate and make decisions that affect peoples’ daily lives (Centers for Disease Control & Prevention, 2011).

Over the past decades, cognitive impairment has been widely recognized as a factor associated with mortality risk among older adults. Across different populations, cognitive impairment increased the risk of mortality by estimates of 18% to 140% (An & Liu, 2016; Iwasa et al., 2013; Lv et al., 2019; Perna et al., 2015; Santabarbara et al., 2015; Takata et al., 2014; Vassilaki et al., 2015). The mortality risk of severe cognitive impairment is higher, compared with people with less cognitive impairment (Perna et al., 2015; Santabarbara et al., 2015). Using participant data from the 1998 wave with follow‐up until the 2011–2012 wave from the Chinese Longitudinal Healthy Longevity Survey (CLHLS), An et al. (An & Liu, 2016) found an inverse association between cognitive impairment and longevity among 7474 oldest‐old without a significant sex difference. However, most of these studies are based on the characteristics of baseline without considering the effects of long‐term time‐varying cognitive impairment in the oldest‐old. In addition, previous studies did not examine the potential differences in the associations by factors such as education and urban‐rural residence among the oldest‐old. And there were few studies to adjust for critical confounders such as health behaviors, leisure activities, and diseases conditions. Therefore, in‐depth analyses with more confounders adjusted of whether such a relationship is modified by these factors are needed.

In addition, research on cognitive subdomain can help us understand which subdomains bring higher mortality risk, and whether the decline of certain submain functions still associates with mortality risk when the overall cognitive performance is normal. Several studies from high‐income countries observed the association between cognitive subdomains and mortality that different subdomains including place orientation and attention were significantly associated with mortality among different population, mostly among adults younger than 80 years old (Iwasa et al., 2013). However, the evidence is inconsistent (Lavery et al., 2009; Park et al., 2013) and the evidence from low‐ and middle‐income countries and the oldest‐old is far from enough. Low‐ and middle‐income countries should be of particular policy focus since these countries are poised to experience the fastest rise in life expectancy. Therefore, understanding cognitive impairment and decline as a predictor of mortality is necessary to illustrate and project morbidity and mortality burdens.

In the present study, we used five waves of the CLHLS (*n* = 25,285) with up to 16.5 years of follow‐up to estimate (i) the association between time‐varying cognitive impairment and all‐cause mortality among the oldest‐old; (ii) the differences of the associations by sex, age group, education attainment, and urban/rural residence; (iii) and the association between cognitive subdomains and all‐cause mortality. Illuminating the disparity among subpopulations and the relationship with cognitive subdomains may help to facilitate future policy making and prevention strategies.

## 2 METHODS

Permission for data use was given by the CLHLS before conducting this study.

### 2.1 Data source

The data were from the CLHLS, initiated in 1998 and followed up in 2000, 2002, 2005, 2008, 2011–2012, and 2014. The study randomly selected half of the counties and cities of 22 out of 30 provinces and was the largest database on the oldest‐old in the world, with survey areas covering 85% of the Chinese population (Zeng, 2012). The CLHLS aims to shed new light on better understanding of determinants of healthy aging. In each wave, survivors were re‐interviewed in the aspects of health status, demographic, socio‐economic, behavioral factors. Deceased participants were substituted by new participants of the same sex and within the same age range of five years. More details of the study can be found in previous publications (Zeng, 2012). Ethic approval was obtained from the Research Ethics Committees of Peking University and Duke University. All survey respondents gave informed consent before participation.

Due to the small number of newly added participants in 2011–2012 (*n* = 1340) and the short follow‐up time to 2013–2014, we only included participants from 1998, 2000, 2002, 2005, and 2008 waves. Among 33,353 participants aged 80 years old or older at enrollment in the five waves, 451 people had missing values on key variables and 7617 people were lost to follow‐up after the baseline. Thus, we pooled 25,285 participants aged 80 or above together in our analyses, among whom 92% participants died during the follow‐up period until 2014. We also included those lost to follow‐up and regarded them as alive or dead in the sensitivity analysis.

Participants enrolled, lost, and died during the follow‐up period by survey were displayed in Figure S1 in the Supporting Information, excluding 451 individuals with missing values. There were 6857, 4498, 3765, 4149, and 6016 individuals in 1998, 2000, 2002, 2005, and 2008 waves, respectively. Among the 25,285 participants and a total of 43,518 times of follow‐up, the duration of follow‐up varied from 0 to 16.5 years, with a median of 2.6 years.

### 2.2 Data on mortality

Information on death was collected from death certificates provided by the local government department. When such information was not available, knowledgeable relatives of the decedents were interviewed. Duration of follow‐up was the time interval from the first interview date until the date of death. Participants who survived the last interview were regarded as being censored on the dates of their last interviews in 2014.

### 2.3 Cognitive impairment

We used time‐varying cognitive impairment, assessed at each wave, as the main exposure in our study. Cognitive impairment was assessed for all participants using the Chinese version of the Mini‐Mental State Exam (MMSE), which was proven to be reliable and valid in previous studies (Chou, 2003; Zhang, 1993). The Chinese version of MMSE evaluates cognitive function through 24 components encompassing seven subdomains: orientation (four points for time orientation, and one point for place orientation); naming foods (naming as many kinds of food as possible in 1 min, seven points); registration of three words (three points); attention and calculation (mentally subtracting three iteratively from twenty, five points); copy a figure (one point); recall (delayed recall of the three words mentioned above, three points); and language (two points for naming objectives, one point for repeating a sentence, and three points for listening and obeying). The MMSE score ranges from 0 to 30. A higher score represents better cognitive function.

Consistent with previous studies (Mungas, 1991), we classified cognitive impairment into four mutually exclusive groups: no cognitive impairment (25 ≤ MMSE score ≤ 30), mild (18 ≤ MMSE score ≤ 24), moderate (10 ≤ MMSE score ≤ 17), and severe (0 ≤ MMSE score ≤ 9) following previous studies (Nguyen et al., 2003; Zhang, 2006). We also used a low cutoff point in this study because a majority of participants were illiterate or had a low education level (Mungas, 1991). In our study, we regarded “unable to answer” as a “wrong answer” (Ashford et al., 1989; Razani et al., 2009). If the participant had missing values or was unable to answer up to 10 components of the MMSE, the data would be regarded as missing and excluded in the following analyses.

Each of the seven cognitive subdomains was dichotomized with full score indicating no impairment and all others as impaired.

### 2.4 Covariates

We considered five groups of covariates in our study: demographic characteristics, health behaviors, leisure activities (Zeng et al., 2010), diseases conditions, and year of the interview. Demographic characteristics included continuous age, sex, education (none, primary school, or middle school or higher), ethnicity (Han vs. minority), place of residence (urban vs. rural), marital status (currently married and living with spouse, separated/divorced/never married, or widowed), occupation before 60 (manual, non‐manual, or professional), and co‐residence (with household members, alone, or in an institution). All demographic variables except for age were regarded as non‐time‐varying variables. Health behaviors included smoking (never, former, or current; measured by asking “do you smoke in the present?” and “did you smoke in the past?”), drinking (never, moderately, or heavy; calculated by the type, frequency, and amount of current drinking consumption (Ge, 2011). Specifically, if a man drinks ≤50 g of liquor containing ≥38° of alcohol, or ≤100 g of liquor containing <38°of alcohol, or ≤250 g fruit wine or rice wine, or ≤750 g beer each day in the present, the moderate drinking was defined; for women, the measurement for moderate drinking was ≤50 g of liquor containing <38°of alcohol, or ≤150 g fruit wine or rice wine, or ≤450 g beer each day. If a person drinks more than that each day, the heavy drinking was defined.), physical activity (yes vs. no; measured by asking “do you take exercise regularly in the present?”), and diet (intake frequency of ten foods: always or almost every day, sometimes or occasionally, or rarely or never).

The frequency of seven leisure activities (e.g., housework, garden work) was measured at each survey, and each activity was classified into “almost every day,” “sometimes,” or “never.” (Ashford et al., 1989) Disease status, which was aggregated by hypertension, diabetes, cardiovascular disease, stroke, chronic obstructive pulmonary disease, and cancer (except for prostate tumor), was categorized into severe, mild, or no disease according to the self‐reported disease history, and the corresponding influence on daily life. Disability was assessed in six activities of daily living (ADL) (dressing, bathing, toileting, feeding, transferring, and continence) (Zeng et al., 2010), and categorized into no ADL limitation if one didn't need any assistance in all six activities, one ADL limitation if one needed any assistance or could not do it at all in at least one of the six activities, or ≥2 ADL limitations for the rest. We also considered year of the interview as a categorical covariate to control the potential period effect.

As health behaviors, leisure activities, and diseases conditions were assessed repeatedly at each wave, they were regarded as time‐varying covariates in this study.

### 2.5 Statistical analysis

We compared characteristics by baseline cognitive impairment status using $\chi^{2}$ test for categorical variables and analysis of variance for continuous variables, and calculated raw mortality by several major demographic factors (age groups, sex, education, and rural/urban residence) and baseline cognitive status. The Kaplan–Meier method was used to graph survival curves by baseline cognitive impairment status and sex. Hazard ratios and 95% confidence intervals (CI) of time‐varying cognitive impairment were calculated using five time‐dependent Cox proportional hazards models: Model 1: adjusted for time‐varying continuous age and sex; Model 2: adjusted for all demographic variables and year of the interview; Model 3: all variables in Model 2 plus time‐varying covariates of health behaviors; Model 4: all variables in Model 3 plus time‐varying leisure activities; and Model 5: all variables in Model 4 plus time‐varying disease status and ADL disability. To decide whether to adjust for the above covariates, we used the “significance‐test‐of‐the‐covariance” strategy, in which a variable is controlled if its coefficient is significantly different from zero at a significance level of 0.05, by adding the covariates in the model one‐by‐one (Maldonado & Greenland, 1993). We tested and ascertained that the proportional hazard assumption had not been violated.

In order to assess disparities across different populations, we also conducted subgroup analyses by age groups (80–89, 90–99, or ≥100 years), sex (male/female), education (none, primary school, or middle school or higher), and residence (urban/rural), respectively. Additionally, we examined the association of subdomain impairment and mortality by including seven subdomains together, among the total population, population without impairment, with mild impairment, and moderate or severe impairment separately. We combined individuals with moderate and severe impairment as the numbers by each subgroup were too small.

CLHLS calculated the corresponding weights at each survey. For example, the weight for a participant interviewed in 1998 was estimated based on the estimated numbers of oldest‐old persons by age, sex, and rural/urban residence in 1998 derived from 2000 census 100% data tabulations for the 22 provinces where the 1998 survey was conducted. The total number of the weighted individual cases of the survey was equal to the total sample size. In the main analysis, we fitted weighted models adjusting for weights in each year of the interview. We also conducted five unweighted models in the sensitivity analysis among all participants.

All estimates were considered statistically significant at *p* < .05. All analyses were performed by SAS version 9.4 (SAS Institute, Inc., Cary, NC) and verified with STATA 13.0 (Stata Corp, College Satiation, TX).

## 3 RESULTS

### 3.1 Descriptive characteristics

We compared the baseline characteristics (Table S1 in the Supporting Information) and time‐varying covariates (Table S2 in the Supporting Information) of included and excluded participants, and found that those excluded tended to be younger, have better socio‐economic status (i.e., better educated, or living in urban areas), have better performance in cognition subdomains and the global cognition function, and healthier lifestyle (fewer current smokers, more non‐drinkers or more persons physically active).

Participants with worse cognitive function (i.e., lower scores on the MMSE) were more likely to be older, female, lower educated, Han ethnicity, living in rural areas, widowed, having had manual occupations, and living with household member(s) (Table 1). Participants were less likely to smoke, drink in the later years of interview, but were more likely to have disease conditions (Table S3 in the Supporting Information).

**TABLE 1:** Baseline characteristics by cognitive function among Chinese aged ≥80 years

	Cognitive impairment^a^					
	Not impaired (*N* = 10,511)	Mild (*N* = 6113)	Moderate (*N* = 3550)	Severe (*N* = 5111)	Total (*N* = 25,285)	*p* value^b^
**Age**, years, median (25th, 75th)	89 (84, 95)	93 (88, 100)	99 (92, 101)	100 (94, 101)	93 (87, 100)	<.001
**Sex**, count (%)						<.001
Male	5588 (53.2)	2005 (32.8)	864 (24.3)	1137 (22.3)	9594 (37.9)	
Female	4923 (46.8)	4108 (47.2)	2686 (75.7)	3974 (77.8)	15,691 (62.1)	
**Education**, count (%)						<.001
None	6111 (58.1)	4786 (78.3)	3059 (86.2)	4375 (85.6)	18,331 (72.5)	
Primary school	2910 (27.7)	1003 (16.4)	372 (10.5)	537 (10.5)	4822 (19.1)	
Middle school or higher	1490 (14.2)	324 (5.3)	119 (3.4)	199 (3.9)	2132 (8.4)	
**Ethnicity**, count (%)						<.001
Han	9725 (92.5)	5668 (92.7)	3324 (93.6)	4866 (95.2)	23,583 (93.3)	
The minority	786 (7.5)	445 (7.3)	226 (6.4)	245 (4.8)	1702 (6.7)	
**Residence**						<.001
Urban	4424 (42.1)	2203 (36.0)	1204 (33.9)	1758 (34.4)	9589 (37.6)	
Rural	6087 (57.9)	3910 (64.0)	2346 (66.1)	3353 (65.6)	15,696 (62.4)	
**Marital status**, count (%)						<.001
Currently married and living with spouse	2465 (23.5)	802 (13.1)	258 (7.3)	321 (6.3)	3846 (15.2)	
Separated/divorced/never married	361 (3.4)	144 (2.4)	63 (1.8)	91 (1.8)	659 (2.6)	
Widowed	7685 (73.1)	5167 (84.5)	3229 (91.0)	4699 (91.9)	20,780 (82.2)	
**Occupation before 60 years**, count (%)						<.001
Manual	9683 (92.1)	5965 (97.6)	3506 (98.8)	5016 (98.1)	24,170 (95.6)	
Non‐manual	330 (3.1)	54 (0.9)	17 (0.5)	30 (0.6)	431 (1.7)	
Professional	498 (4.7)	94 (1.5)	27 (0.8)	65 (1.3)	684 (2.7)	
**Co‐residence**, count (%)						<.001
With household member(s)	8612 (81.6)	5041 (82.5)	3055 (86.1)	4558 (89.2)	21,266 (84.1)	
Alone	1500 (14.3)	836 (13.7)	388 (10.9)	406 (7.9)	3130 (12.4)	
In an institution	399 (3.8)	236 (3.9)	107 (3.0)	147 (2.9)	889 (3.5)	

^a^ Measured by the Chinese version of the Mini‐Mental State Exam (MMSE) and classified into no cognitive impairment (25 ≤ MMSE score ≤ 30), mild (18 ≤ MMSE score ≤ 24), moderate (10 ≤ MMSE score ≤ 17), and severe (0 ≤ MMSE score ≤ 9).

^b^ Chi‐square test was used for categorical variables, and analysis of variance was used for continuous variables.

As expected, participants with advanced age and lower education level had higher raw mortality rate, although the trend was not necessarily applied to participants with different cognitive statuses (Table S4 in the Supporting Information). Females had higher raw mortality in the overall population or subpopulation grouped by cognitive status than male. Urban and rural residents turned to have similar overall raw mortality rates.

### 3.2 Kaplan–Meier curves and results of multivariable analysis among all participants

**FIGURE 1:**
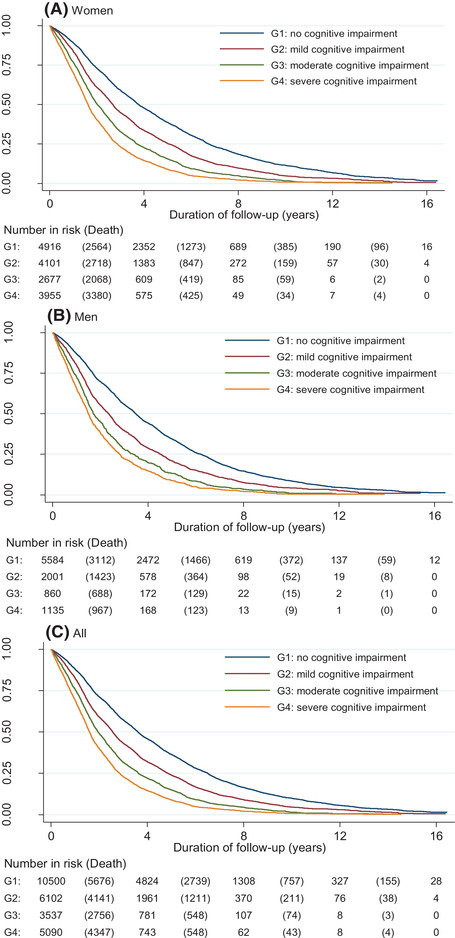
Kaplan–Meier survival curves by baseline cognitive impairment status and sex for (a) women, (b) men, and (c) all

Kaplan–Meier survival curves illustrated a clear dose–response relationship between the severity of baseline cognitive impairment and lower probability of survival for both female and male and total population (log‐rank test for trend: all *p*‐value <.001, Figure 1). The median survival time of no, mild, moderate, and severe cognitive impairment were 3.6, 2.6, 1.9, and 1.6 years, respectively, among the total population.

**FIGURE 2:**
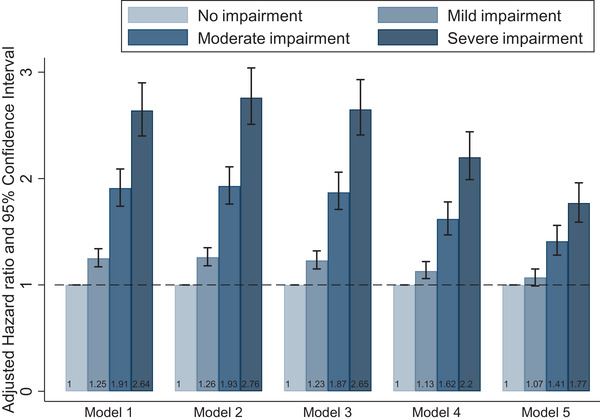
Multivariable‐adjusted hazard ratios and 95% confidence intervals of all‐cause mortality by cognitive function among Chinese aged ≥80 years † Measured by the Chinese version of the Mini‐Mental State Exam (MMSE) and classified into no cognitive impairment (25≤ MMSE score ≤30), mild (18≤ MMSE score ≤24), moderate (10≤ MMSE score ≤17), and severe (0≤ MMSE score ≤9). Model 1: adjusted for continuous age and sex. Model 2: adjusted for education, ethnicity, residence, marital status, and year of interview based on model 1. Model 3: adjusted for smoking, drinking, physical activity, and diet (vegetable, egg, and garlic) based on model 2. Model 4: adjusted for leisure activities (housework, field work, garden work, reading newspaper, raising pets, playing mahjong/cards, and watching TV or listening to videos) based on model 3. Model 5: adjusted for disease status and ADL disability based on model 4.

The first four multivariate models displayed consistent dose–response patterns between time‐varying cognitive impairment and mortality, and the association attenuated with more covariates in the models (Figure 2). After adjustment of health behaviors, leisure activities, disease status, and ADL disability in Model 5, moderate (HR = 1.41, 95% CI 1.28–1.56) and severe (HR = 1.77, 95% CI 1.59–1.96) cognitive impairment were significantly associated with increased mortality, compared to no cognitive impairment. Mildly impaired participants showed no different death risk than those unimpaired, although the dose–response pattern still existed irrespective of the insignificance of mild impairment. One unit decrease in MMSE score was associated with increased mortality with a hazard ratio of 1.013 (95% CI 1.013–1.016).

### 3.3 Subgroup analyses

**FIGURE 3:**
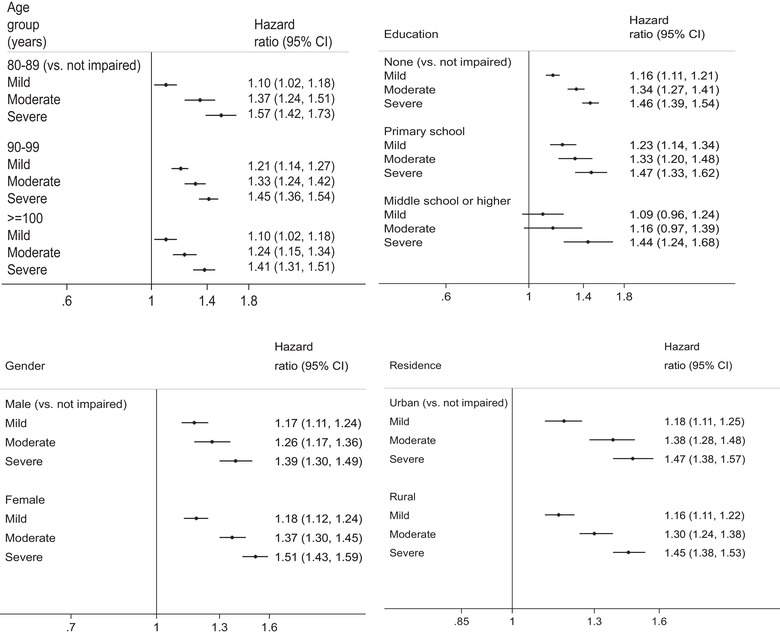
Multivariable‐adjusted hazard ratios and 95% confidence intervals of all‐cause mortality by cognitive function *in subgroups* among Chinese aged ≥80 years † Abbreviation: CI, confidence interval. † All models were adjusted for demographics (continuous age, sex, education, ethnicity, residence, and marital status) except for the corresponding factor in each subgroup model, and smoking, drinking, physical activity, diet (vegetable, egg, and garlic), leisure activities (house work, field work, garden work, reading newspaper, raising pets, playing mahjong/cards, and watching TV or listening to videos), disease status, ADL disability and year of interview. Continuous age was adjusted in the age group models.

**FIGURE 4:**
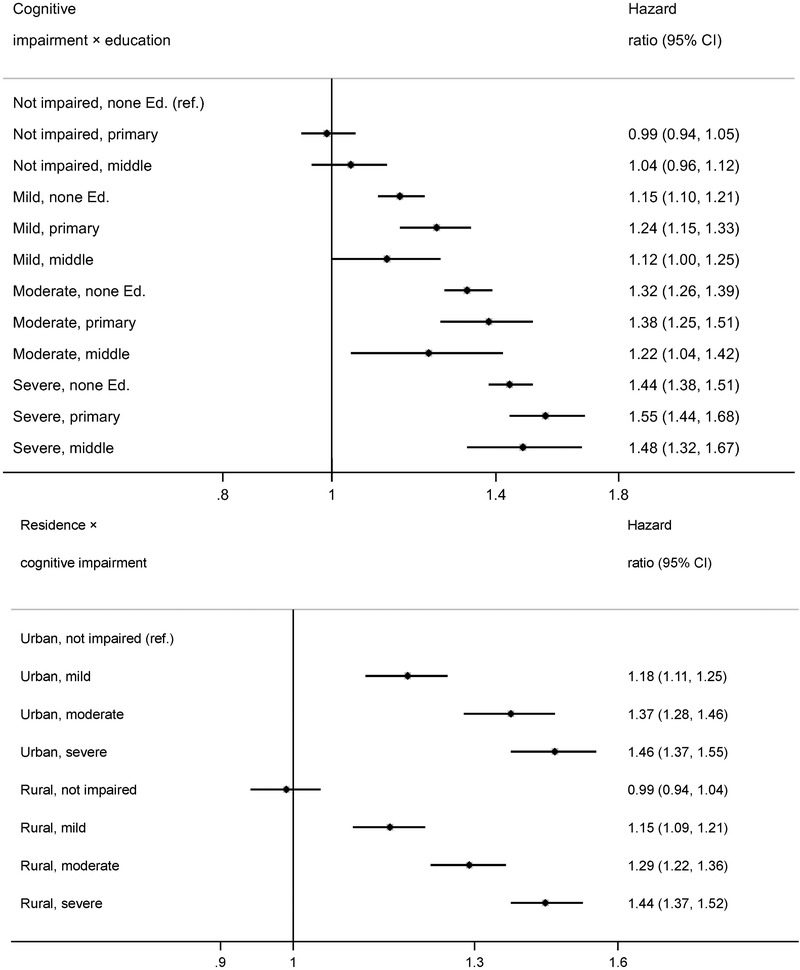
Interactions between cognitive impairment and education, residence among Chinese aged ≥80 years † Abbreviation: CI, confidence interval. † All models were adjusted for demographics (age, sex, education, ethnicity, residence, and marital status) except for the corresponding factor in each subgroup model, and smoking, drinking, physical activity, diet (vegetable, egg, and garlic), leisure activities (housework, field work, garden work, reading newspaper, raising pets, playing mahjong/cards, and watching TV or listening to videos), disease status, ADL disability and year of interview.

Figure 3 shows the hazard ratios by cognitive impairment for different subgroups, conducted as separate models for each subgroup with full adjustment as in Model 5. The dose–response relationship and the effect sizes between cognitive impairment and mortality were consistent across all subgroups by age group, sex, urban/rural residence, and education with the exception of the highest education group (middle school or higher). To examine whether the association between cognition and mortality is modified by education, we further tested the interaction between cognitive impairment and education (Table S5 in the Supporting Information, Figure 4). The results showed an insignificant interaction between cognitive impairment and education (*p* = .223). Participants with primary education showed higher mortality than the lowest education group in the cognitively impaired groups (Figure 4).

In order to assess whether mortality risks by cognitive impairment differed by urban/rural residence, we ran one model with all participants stratified into eight subgroups by residence and four impairment groups (Figure 4). We did not find any significant differences by residence; in other words, rural participants had a similar risk of mortality as urban participants with the same level of cognitive impairment.

### 3.4 Subdomain analyses

We examined the seven subdomains separately for the total population and by three cognitive impairment groups (Table 2). Impairment in orientation, naming foods, attention, and calculation, copy figure, and delayed recall was associated with significantly increased mortality risk in the total population, with hazard ratios ranging from 1.17 (naming foods, delayed recall) to 1.33 (orientation). Among those with no cognitive impairment overall (i.e., total MMSE score ≥25), those who had impairment in orientation and copy figure had higher mortality risks than those who were not impaired in these two subdomains. Among mildly impaired, only orientation impairment had significantly higher risks; and among moderately or severely impaired, only orientation and language.

**TABLE 2:** Multivariable‐adjusted Hazard Ratios and 95% confidence intervals of all‐cause mortality by cognitive function subdomain^a^ among Chinese aged ≥80 years^b^

	Total	No impairment	Mild	Moderate and severe
	*n* = 42,084	*n* = 17,699	*n* = 10,259	*n* = 14,126
	Adjusted hazard ratio (95% confidence interval)			
Orientation^c^	**1.33 (1.25, 1.41)**	**1.20 (1.05, 1.36)**	**1.14 (1.01, 1.28)**	**1.23 (1.03, 1.46)**
Naming foods^c^	**1.17 (1.10, 1.23)**	1.08 (0.99, 1.18)	0.99 (0.89, 1.11)	0.95 (0.80, 1.14)
Registration^c^	**1.22 (1.15, 1.30)**	1.10 (0.98, 1.23)	0.98 (0.87, 1.1)	1.07 (0.91, 1.27)
Attention and calculation^c^	**1.19 (1.13, 1.27)**	1.06 (0.96, 1.17)	1.02 (0.90, 1.16)	1.13 (0.84, 1.53)
Copy figure^c^	**1.15 (1.07, 1.24)**	**1.12 (1.03, 1.22)**	0.97 (0.79, 1.18)	0.93 (0.63, 1.36)
Delayed recall^c^	**1.17 (1.11, 1.24)**	1.07 (0.98, 1.16)	0.98 (0.86, 1.1)	1.02 (0.79, 1.33)
Language^c^	**1.21 (1.14, 1.29)**	1.02 (0.91, 1.14)	1.06 (0.94, 1.19)	**1.44 (1.16, 1.79)**

^a^ Measured by the Chinese version of the Mini‐Mental State Exam (MMSE), and each of the seven cognitive subdomains was dichotomized with full score indicating no impairment and all others as impaired.

^b^ Each model was adjusted for all the covariates, including demographics (age, sex, education, ethnicity, residence, and marital status), and smoking, drinking, physical activity, diet (vegetable, egg, and garlic), leisure activities (housework, fieldwork, garden work, reading newspaper, raising pets, playing mahjong/cards, and watching TV or listening to videos), disease status, ADL disability and year of the interview.

^c^ Impaired versus not impaired, and not impaired as the reference group.

### 3.5 Sensitivity analyses

Unweighted models showed a consistent dose–response pattern in all five models (Figure S2 in the Supporting Information). The magnitude of the estimates was smaller compared to the weighted results.

We included those lost to follow‐up and regarded them as alive or dead in the sensitivity analysis (Table S6 in the Supporting Information), and similar results can be found compared to the main analyses in which individuals lost to follow‐up were excluded (Figure 2, Model 5).

## 4 DISCUSSION

In this longitudinal study with up to 16.5 years of follow‐up among the 25,825 adults, 80 years or older in China, we observed a dose–response relationship between MMSE score and risk of mortality. In the overall analysis, the hazard ratio of death was 1.41 for moderately impaired and 1.77 for severely impaired compared to those who were not cognitively impaired, even after adjusting for a large number of covariates including diseases and daily functioning. This relationship was highly consistent across several factors including sex, age group, urban/rural residence, and education. It varied by cognitive subdomain with orientation being the most pronounced in its association with mortality.

Our main finding of higher mortality risk with higher degrees of cognitive impairment was generally consistent with previous studies in Denmark (Andersen et al., 2002), Japan (Iwasa et al., 2013), and China (An & Liu, 2016). However, studies reported inconsistent association between cognition and survival across different age groups. In a multicenter study of a population aged 65+ years old, the association between cognition and mortality was not observed in male aged 85 years and older (Neale et al., 2001). The insignificant association was also found in another population with the mean age of 83.8 years (Matusik et al., 2012). A Japanese study among individuals aged 85+ years old found that 1‐point increase in the global MMSE score increased all‐cause mortality by 4.3% without adjustment (Takata et al., 2014). Using the CLHLS, Lv et al. (Lv et al., 2019) reported that relatively younger older people (aged 65–79 years) had a more pronounced association between rate of change in MMSE and mortality compared with the oldest‐old (>= 80), but the association did not differ by age for categorical cognition. Our findings also yielded a hazard ratio of 1.015 with a 1‐point increase of MMSE in the fully adjusted model. Additionally, we found a consistent dose–response pattern in those aged 80–90 years, 90–100 years, and 100+ years, suggesting the importance of cognitive function among the very oldest‐old.

Cognition was reported to be associated with cardiovascular mortality and senility mortality in the above Japanese study (Takata et al., 2014). The cognitive impairment most likely interacts with the deterioration of somatic function, behavioral and social‐economic factors, and health service utilization to affect death. Using the CLHLS survey data and genotype data from 877 individuals aged 90 years and above, Zeng and colleagues (Zeng, 2012) found the interaction effects of negative emotion, physical exercise, leisure activities, and carrying the rs1042718 minor allele, indicating the necessity to consider a comprehensive profile of individuals’ social, physical, physiological, and genetic factors.

The larger estimates of the weighted models compared to the unweighted models may be attributable to variance inflation due to using sample weights (Korn & Graubard, 1995). Our study reported vanished significance of mild impairment, inconsistent with other studies (An & Liu, 2016; Vassilaki et al., 2015). This could be partially determined by the study methodology (e.g., assessment criteria used, follow‐up time, covariates adjustment, and statistical models) and study population (e.g., age, ethnic, comorbidities) (Vassilaki et al., 2015).

The irregular association between cognition and mortality among the highest educated group may be due to the small sample size in our study. Second, as explicit above, we used a low cutoff score to separate the MMSE score into four groups to accommodate participants’ low education in the CLHLS (Takata et al., 2014). For participants with a higher education level, this method may not be applicable, suggesting different measuring methods of cognition function by education attainment may be warranted.

Furthermore, in China, large urban‐rural disparities exist in terms of socio‐economic development, access to health care resources, and many health outcomes with urban residents enjoying better access to resources (Liu et al., 1999). However, we did not find that rural residents had higher risks of mortality than urban counterparts with the same level of cognitive functioning. We speculate that the similar association between cognitive impairment and mortality in rural and urban areas may reveal the lack of investment in cognition‐ or dementia‐related health care around the country. A large number of elderly people are expected to suffer varying degrees of cognitive impairment in the future (United Nations, Department of Economic & Social Affairs, Population Division 2017). Our findings along with evidence on the lack of adequate care for cognitive impairment in China indicate that there is an urgent need for China's health care system and government to improve provision and quality of these services, if there is a causal relationship between cognitive function and mortality. In any case, those with more severe cognitive impairments can be screened to better predict adverse outcomes and can be targeted for intervention.

Among individuals without overall cognitive impairment, impairment in orientation was significantly associated with raised mortality risk, consistent with a Japanese study (Iwasa et al., 2013). This finding suggests that the orientation subdomain is associated with mortality risk independent of the overall MMSE score. Disorientation might be a risk of getting lost, thus increases mortality risk (Passini et al., 2000). Previous study found that orientation was more impaired in moderate stage of dementia compared to mild stage (Wang et al., 2004), and if combined with memory test, orientation might improve the screening for dementia (Tsai et al., 2004). However, delayed recall that was significantly associated with mortality risk in Japanese study (Iwasa et al., 2013) was no longer statistically significant in this study while we found copy figure was a subdomain with significant association among older adults without overall cognitive impairment. The lower capacity of imitating drawing in China is possibly due to the lower education attainment of the participants. These results are especially interesting for future research studies to identify which neurological or cognitive reserve is more predictive of mortality. It has public health implication that in low‐resource settings without formal MMSE tests, perhaps simple assessments could serve as robust proxies. Further studies focusing on cognition subdomain research in low‐resource populations should be developed to provide more informative evidence for public health implication.

Our study has its own strengths and limitations. Our database was among the few in the world on the oldest‐old population. The large sample size and the long follow‐up period enabled us to conduct detailed subgroup and subdomain analyses with adjustment for a large number of covariates. However, our cognitive measurement depended on the MMSE, albeit validated in population‐based studies (Chou, 2003; Zhang, 1993), is not a professional diagnosis of cognitive impairment. In addition, we did not categorize cognitive impairment tailored to different education levels in the present study. Secondly, we did not have data on causes of death, limiting our ability to do cause‐specific analyses. Lastly, disease history, one of the many adjusted variables, was ascertained by self‐report, which may suffer from inaccuracy and recall bias.

In conclusion, our findings confirmed the consistent dose–response associations between cognitive impairment and mortality in overall population and specific subpopulations using the largest cohort of community‐dwelling oldest‐old adults in China. The oldest‐old is among the fastest growing segment of Chinese population and prevalence of cognitive impairment is high among this age group. Our findings join other studies in calling for actions to improve cognitive functioning of older adults to reduce health risks and improve longevity.

## CONFLICT OF INTEREST

The authors declare no conflicts of interest.

## AUTHOR CONTRIBUTIONS

Yaxi Li and Heng Jiang analyzed the data, drafted the paper, and revised the manuscript. Xurui Jin, Huali Wang, John S. Ji, and Lijing L. Yan revised the manuscript. Lijing L. Yan designed the project, supervised the data analysis, and reviewed the literature.

## SPONSOR'S ROLE

The sponsor had no role in the design, methods, data collections, analysis, or preparation of paper.

RESPONSE TO PEER REVIEW TRANSPARENCY OPTION (reviewer reports, author responses, and decision letter linked from Publons): Agree

### PEER REVIEW

The peer review history for this article is available at https://publons.com/publon/10.1002/brb3.2325

## Supporting information




SUPPORTING INFORMATION

## Data Availability

The CLHLS data is publicly available. Information about the data can befound at http://opendata.pku.edu.cn/dataset.xhtml?persistentId=doi:10.18170/DVN/XRV2WN.
