# Supplementary material for: Cognitive impairment and all‐cause mortality among Chinese adults aged 80 years or older
Source: Brain Behav. 2021 Sep 7;11(10):e2325. doi: 10.1002/brb3.2325 (PMC8553308; doi:10.1002/brb3.2325)
Supplement: Supplementary file 1 — SUPPORTING INFORMATION [file BRB3-11-e2325-s001.docx]

**Appendices:**

**Table S1. Comparison of baseline characteristics between included and excluded participants**

|  | **Included  (n=25,285)** | **Excluded** | | | **p-value**† |
| --- | --- | --- | --- | --- | --- |
|  |  | **Loss to follow-up**  **(n=7,734)** | **Missing key covariates**  **(n= 334)** | **All**  **(n=8,068)** |  |
| **Age**, years | 93 (87, 100) | 91 (85, 99) | 94 (87, 101) | 91 (85, 99) | <.001 |
| **Gender** |  |  |  |  | 0.054 |
| Male | 9,594 (37.9) | 3,037 (39.3) | 121 (36.2) | 3,158 (39.1) |  |
| Female | 15,691 (62.1) | 4,697 (60.7) | 213 (63.8) | 4,910 (60.9) |  |
| **Education** |  |  |  |  | <.001 |
| None | 18,331 (72.5) | 4,955 (64.1) | 126 (37.7) | 5,081 (63.0) |  |
| Primary school | 4,822 (19.1) | 1,571 (20.3) | 37 (11.1) | 1,608 (19.9) |  |
| Middle school or higher | 2,132 (8.4) | 1,147 (14.8) | 13 (3.9) | 1,160 (14.4) |  |
| Missing | 0 | 61 (0.8) | 158 (47.3) | 219 (2.7) |  |
| **Ethnicity** |  |  |  |  | <.001 |
| Han | 23,583 (93.3) | 235 (3) | 7 (2.1) | 7,792 (96.6) |  |
| The Minority | 1,702 (6.7) | 7,484 (96.8) | 308 (92.2) | 242 (3.0) |  |
| Missing | 0 | 15 (0.2) | 19 (5.7) | 34 (0.4) |  |
| **Residence** |  |  |  |  | <.001 |
| Urban | 9,589 (37.9) | 4,571 (59.1) | 156 (46.7) | 4,727 (58.6) |  |
| Rural | 15,696 (62.1) | 3,163 (40.9) | 178 (53.3) | 3,341 (41.4) |  |
| **Marital** **status** |  |  |  |  | <.001 |
| Currently married and living with spouse | 3,846 (15.2) | 1,456 (18.8) | 39 (11.7) | 1,495 (18.5) |  |
| Separated/divorced/never married | 659 (2.6) | 211 (2.7) | 17 (5.1) | 228 (2.8) |  |
| Widowed | 20,780 (82.2) | 6,064 (78.4) | 273 (81.7) | 6,337 (78.5) |  |
| Missing | 0 | 3 (0) | 5 (1.5) | 8 (0.1) |  |
| **Occupation** |  |  |  |  | <.001 |
| Manual | 24,170 (95.6) | 6,989 (90.4) | 261 (78.1) | 7,250 (89.9) |  |
| Non-manual | 431 (1.7) | 275 (3.6) | 4 (1.2) | 279 (3.5) |  |
| Professional | 684 (2.7) | 454 (5.9) | 9 (2.7) | 463 (5.7) |  |
| Missing | 0 | 16 (0.2) | 60 (18) | 76 (0.9) |  |
| **Co-residence** |  |  |  |  | <.001 |
| With household member(s) | 21,266 (84.1) | 6,176 (79.9) | 249 (74.6) | 6,425 (79.6) |  |
| Alone | 3,130 (12.4) | 1,118 (14.5) | 46 (13.8) | 1,164 (14.4) |  |
| In an institution | 889 (3.5) | 440 (5.7) | 37 (11.1) | 477 (5.9) |  |
| Missing | 0 | 0 (0) | 2 (0.6) | 2 (0.0) |  |
| **Years of enrolling** |  |  |  |  | <.001 |
| 1998 | 6,857 (27.1) | 1,970 (25.5) | 132 (39.5) | 2,102 (26.1) |  |
| 2000 | 4,498 (17.8) | 1,764 (22.8) | 75 (22.5) | 1,839 (22.8) |  |
| 2002 | 3,756 (14.9) | 1,013 (13.1) | 81 (24.3) | 1,094 (13.6) |  |
| 2005 | 4,149 (16.4) | 1,370 (17.7) | 23 (6.9) | 1,393 (17.3) |  |
| 2008 | 6,016 (23.8) | 1,617 (20.9) | 23 (6.9) | 1,640 (20.3) |  |

†Characteristics comparison for included and excluded individuals.

**Table S2. Comparison of time-varying characteristics between included and excluded participants**

|  | **Included  (n=43,639)** | **Excluded** | | | **p-value** † |
| --- | --- | --- | --- | --- | --- |
|  |  | **Loss to follow-up**  **(n=12,139)** | **Missing key covariates**  **(n= 733)** | **All**  **(n=12,872)** |  |
| **Orientation** |  |  |  |  | <.001 |
| Deficiency | 18,406 (42.2) | 4,443 (36.6) | 344 (46.9) | 4,787 (37.2) |  |
| Integrity | 25,233 (57.8) | 7,672 (63.2) | 340 (46.4) | 8,012 (62.2) |  |
| Missing | 0 | 24 (0.2) | 49 (6.7) | 73 (0.6) |  |
| **Naming** **Foods** |  |  |  |  | <.001 |
| Deficiency | 24,190 (55.4) | 5,914 (48.7) | 407 (55.5) | 6,321 (49.1) |  |
| Integrity | 19,449 (44.6) | 6,201 (51.1) | 277 (37.8) | 6,478 (50.3) |  |
| Missing | 0 | 24 (0.2) | 49 (6.7) | 73 (0.6) |  |
| **Registration** |  |  |  |  | <.001 |
| Deficiency | 19,945 (45.7) | 4,707 (38.8) | 364 (49.7) | 5,071 (39.4) |  |
| Integrity | 23,694 (54.3) | 7,408 (61) | 320 (43.7) | 7,728 (60) |  |
| Missing | 0 | 24 (0.2) | 49 (6.7) | 73 (0.6) |  |
| **Attention** **and** **Calculation** |  |  |  |  | <.001 |
| Deficiency | 25,010 (57.3) | 6,020 (49.6) | 442 (60.3) | 6,462 (50.2) |  |
| Integrity | 18,629 (42.7) | 6,095 (50.2) | 242 (33) | 6,337 (49.2) |  |
| Missing | 0 | 24 (0.2) | 49 (6.7) | 73 (0.6) |  |
| **Copy** **Figure** |  |  |  |  | <.001 |
| Deficiency | 36,974 (84.7) | 9,450 (77.9) | 586 (80) | 10,036 (78) |  |
| Integrity | 6,665 (15.3) | 2,665 (22) | 98 (13.4) | 2,763 (21.5) |  |
| Missing | 0 | 24 (0.2) | 49 (6.7) | 73 (0.6) |  |
| **Delayed** **recall** |  |  |  |  | <.001 |
| Deficiency | 25,912 (59.4) | 6,546 (53.9) | 441 (60.2) | 6,987 (54.3) |  |
| Integrity | 17,727 (40.6) | 5,569 (45.9) | 243 (33.2) | 5,812 (45.2) |  |
| Missing | 0 | 24 (0.2) | 49 (6.7) | 73 (0.6) |  |
| **Language** |  |  |  |  | <.001 |
| Deficiency | 22,457 (51.5) | 5,389 (44.4) | 394 (53.8) | 5,783 (44.9) |  |
| Integrity | 21,182 (48.5) | 6,726 (55.4) | 290 (39.6) | 7,016 (54.5) |  |
| Missing | 0 | 24 (0.2) | 49 (6.7) | 73 (0.6) |  |
| **Cognition Impairment** |  |  |  |  | <.001 |
| No | 18,042 (41.3) | 6,050 (49.8) | 228 (31.1) | 6,278 (48.8) |  |
| Mild | 10,599 (24.3) | 2,816 (23.2) | 172 (23.5) | 2,988 (23.2) |  |
| Moderate | 6,111 (14) | 1,413 (11.6) | 78 (10.6) | 1,491 (11.6) |  |
| Severe | 8,887 (20.4) | 1,836 (15.1) | 206 (28.1) | 2,042 (15.9) |  |
| Missing | 0 | 24 (0.2) | 49 (6.7) | 73 (0.6) |  |
| **Smoking** |  |  |  |  | <.001 |
| Current | 6,770 (15.5) | 1,648 (13.6) | 81 (11.1) | 1,729 (13.4) |  |
| Former | 7,035 (16.1) | 2,102 (17.3) | 95 (13) | 2,197 (17.1) |  |
| Never | 29,834 (68.4) | 8,357 (68.8) | 452 (61.7) | 8,809 (68.4) |  |
| Missing | 0 | 32 (0.3) | 105 (14.3) | 137 (1.1) |  |
| **Drinking** |  |  |  |  | <.001 |
| Never | 35,260 (80.8) | 10,177 (83.8) | 462 (63) | 10,639 (82.7) |  |
| Moderate | 4,396 (10.1) | 1,013 (8.4) | 52 (7.1) | 1,065 (8.3) |  |
| Heavy | 3,983 (9.1) | 902 (7.4) | 44 (6) | 946 (7.4) |  |
| Missing | 0 | 47 (0.4) | 175 (23.9) | 222 (1.7) |  |
| **Physical activity** |  |  |  |  | <.001 |
| No | 32,678 (74.9) | 8,139 (67.1) | 540 (73.7) | 8,679 (67.4) |  |
| Yes | 10,961 (25.1) | 3,990 (32.9) | 154 (21) | 4,144 (32.2) |  |
| Missing | 0 | 10 (0.1) | 39 (5.3) | 49 (0.4) |  |

†Characteristics comparison for included and excluded individuals.

**Table S3. Time-varying characteristics by year of interview among Chinese aged ≥80 years**

|  | **Year of interview** | | | | | | |  |
| --- | --- | --- | --- | --- | --- | --- | --- | --- |
| **Characteristics** | **1998**  **(N=** **6,857)** | **2000**  **(N=** **8,046)** | **2002**  **(N=** **8,500)** | **2005**  **(N=** **7,448)** | **2008**  **(N=** **8,906)** | **2011**  **(N=** **3,761)** | **Total**  **(N= 43,518)** | ***P* value** |
| **Cognition Impairment** |  |  |  |  |  |  |  | <0.001 |
| No | 3,126 (45.6) | 3,950 (49.1) | 3,287 (38.7) | 2,979 (40) | 3,146 (35.3) | 1,517 (40.3) | 18,005 (41.4) |  |
| Mild | 1,718 (25.1) | 1,890 (23.5) | 2,246 (26.4) | 1,705 (22.9) | 2,141 (24) | 869 (23.1) | 10,569 (24.3) |  |
| Moderate | 931 (13.6) | 946 (11.8) | 1,303 (15.3) | 1,111 (14.9) | 1,271 (14.3) | 530 (14.1) | 6,092 (14.0) |  |
| Severe | 1,082 (15.8) | 1,260 (15.7) | 1,664 (19.6) | 1,653 (22.2) | 2,348 (26.4) | 845 (22.5) | 8,852 (20.3) |  |
| **Smoking**, Count (%) |  |  |  |  |  |  |  |  |
| Current | 1,175 (17.1) | 1,399 (17.4) | 1,273 (15) | 1,117 (15.0) | 1,258 (14.1) | 529 (14.1) | 6,751 (15.5) | <0.001 |
| Former | 1,010 (14.7) | 1,386 (17.2) | 1,468 (17.3) | 1,221 (16.4) | 1,318 (14.8) | 612 (16.3) | 7,015 (16.1) |  |
| Never | 4,672 (68.1) | 5,261 (65.4) | 5,759 (67.8) | 5,110 (68.6) | 6,330 (71.1) | 2,620 (69.7) | 29,752 (68.4) |  |
| **Drinking**, Count (%) |  |  |  |  |  |  |  |  |
| Never | 5,203 (75.9) | 6,357 (79) | 6,827 (80.3) | 6,059 (81.4) | 7,486 (84.1) | 3,224 (85.7) | 35,156 (80.8) | <0.001 |
| Moderate | 751 (11) | 894 (11.1) | 880 (10.4) | 776 (10.4) | 796 (8.9) | 295 (7.8) | 4,392 (10.1) |  |
| Heavy | 903 (13.2) | 795 (9.9) | 793 (9.3) | 613 (8.2) | 624 (7) | 242 (6.4) | 3,970 (9.1) |  |
| **Physical activity**, Count (%) |  |  |  |  |  |  |  |  |
| Yes | 5,274 (76.9) | 5,576 (69.3) | 6,259 (73.6) | 5,702 (76.6) | 7,074 (79.4) | 2,696 (71.7) | 32,581 (74.9) | <0.001 |
| No | 1,583 (23.1) | 2,470 (30.7) | 2,241 (26.4) | 1,746 (23.4) | 1,832 (20.6) | 1,065 (28.3) | 10,937 (25.1) |  |
| **Disease**, Count (%) |  |  |  |  |  |  |  |  |
| Severe | 363 (5.3) | 567 (7.1) | 765 (9) | 727 (9.8) | 952 (10.7) | 378 (10.1) | 3,752 (8.6) | <0.001 |
| Mild | 1,768 (25.8) | 1,943 (24.2) | 2,122 (25) | 1,889 (25.4) | 2,017 (22.7) | 1,232 (32.8) | 10,971 (25.2) |  |
| No diseases | 4,379 (63.9) | 5,253 (65.3) | 5,401 (63.5) | 4,579 (61.5) | 5,873 (65.9) | 2,041 (54.3) | 27,526 (63.3) |  |
| Missing or don’t know | 347 (5.1) | 283 (3.5) | 212 (2.5) | 253 (3.4) | 64 (0.7) | 110 (2.9) | 1,269 (2.9) |  |
| **Activity of daily living (ADL)**, Count (%) |  |  |  |  |  |  |  |  |
| No ADL limitation | 4,141 (60.4) | 5,151 (64) | 4,967 (58.4) | 4,792 (64.3) | 6,276 (70.5) | 2,244 (59.7) | 27,571 (63.4) | <0.001 |
| One ADL limitation | 913 (13.3) | 1,130 (14) | 1,429 (16.8) | 1,020 (13.7) | 940 (10.6) | 551 (14.7) | 5,983 (13.8) |  |
| $\geq$2 ADL limitations | 1,803 (26.3) | 1,765 (21.9) | 2,104 (24.8) | 1,636 (22) | 1,690 (19) | 966 (25.7) | 9,964 (22.9) |  |

**Table S4. The raw mortality rate** † **by characteristics and baseline cognitive function among Chinese aged ≥80 years**

|  | **Cognitive Impairment** † | | | |  |
| --- | --- | --- | --- | --- | --- |
|  | **Not impaired** | **Mild** | **Moderate** | **Severe** | **Total** |
|  | **(N=10,511)** | **(N= 6,113)** | **(N= 3,550)** | **(N=5,111)** | **(N=25,285)** |
| **Age group** |  |  |  |  |  |
| 80~ | 164 (159, 169) | 189 (180, 199) | 250 (229, 272) | 301 (273, 332) | 179 (174, 183) |
| 90~ | 256 (247, 264) | 309 (296, 322) | 360 (341, 380) | 437 (416, 459) | 307 (301, 314) |
| 100~ | 337 (320, 354) | 387 (370, 405) | 442 (421, 465) | 507 (490, 526) | 423 (414, 432) |
| **Sex** |  |  |  |  |  |
| Male | 197 (191, 203) | 270 (261, 279) | 359 (345, 373) | 455 (441, 469) | 263 (257, 268) |
| Female | 218 (212, 225) | 303 (289, 317) | 399 (373, 428) | 473 (446, 502) | 281 (277, 286) |
| **Education** |  |  |  |  |  |
| None | 209 (203, 214) | 274 (266, 282) | 363 (350, 377) | 452 (439, 466) | 285 (281, 290) |
| Primary school | 208 (200, 216) | 300 (282, 320) | 425 (384, 472) | 504 (463, 550) | 252 (245, 260) |
| Middle school or higher | 206 (195, 217) | 311 (278, 348) | 344 (286, 415) | 485 (421, 559) | 238 (227, 248) |
| **Residence** |  |  |  |  |  |
| Urban | 208 (202, 215) | 296 (284, 309) | 394 (372, 418) | 481 (459, 504) | 274 (268, 280) |
| Rural | 208 (202, 213) | 271 (262, 280) | 356 (341, 371) | 448 (432, 463) | 274 (270, 279) |

† The unit is per 1,000 person years.

**Table S5. Interaction between cognition function and education among Chinese aged ≥80 years**

|  | Model 1 † | Model 2 † | Model 3 † |
| --- | --- | --- | --- |
|  | Adjusted hazard ratio (95% confidence interval) | | |
| **Cognitive impairment** |  |  |  |
| Not impaired | Ref. | Ref. | Ref. |
| Mild | 1.17 (1.12, 1.21) | 1.17 (1.13, 1.21) | 1.15 (1.1, 1.21) |
| Moderate | 1.33 (1.27, 1.38) | 1.33 (1.27, 1.39) | 1.32 (1.26, 1.39) |
| Severe | 1.46 (1.4, 1.52) | 1.46 (1.4, 1.53) | 1.44 (1.38, 1.51) |
| **Education** |  |  |  |
| None |  | Ref. | Ref. |
| Primary school |  | 1.03 (1, 1.07) | 0.99 (0.94, 1.05) |
| Middle school or higher |  | 1.02 (0.96, 1.07) | 1.04 (0.96, 1.12) |
| **Cognitive impairment** × **Education** |  | Wald test of interaction (6) = 8.22, P=0.223 | |
| Not impaired, no education |  |  | Ref. |
| Mild, primary school |  |  | 1.08 (0.99, 1.18) |
| Mild, middle school |  |  | 0.93 (0.82, 1.06) |
| Moderate, primary school |  |  | 1.05 (0.94, 1.17) |
| Moderate, middle school |  |  | 0.88 (0.74, 1.05) |
| Severe, primary school |  |  | 1.08 (0.99, 1.19) |
| Severe, middle school |  |  | 0.99 (0.87, 1.13) |

† Adjusted for age, sex, ethnicity, residence, marital status, smoking, drinking, physical activity, diet (vegetable, egg, and garlic), leisure activities (housework, fieldwork, garden work, reading newspaper, raising pets, playing mahjong/cards, and watching TV or listening to videos), disease status, ADL disability and year of the interview.

**Table S6.** **Multivariable-adjusted hazard ratios and 95% confidence intervals of all-cause mortality by cognitive function among Chinese aged ≥80 years including individuals lost to follow up. Individuals lost to follow up were assumed to be alive or dead but lost to follow up in the middle time of surveyed years.** †

| **Subgroup** | **Cognitive impairment** | | | |
| --- | --- | --- | --- | --- |
|  | **No** | **Mild** | **Moderate** | **Severe** |
|  | **Adjusted Hazard ratio and 95% CI in subgroups** | | | |
| **Assumed those lost to follow up to be alive** | | | | |
| Male | Ref. | 1.10 (0.98, 1.23) | 1.22 (1.03, 1.46) | 1.6 (1.33, 1.92) |
| Female | Ref. | 1.04 (0.95, 1.14) | 1.54 (1.38, 1.73) | 1.9 (1.68, 2.17) |
| Total | Ref. | 1.06 (0.99, 1.14) | 1.44 (1.31, 1.58) | 1.79 (1.62, 1.99) |
| **Assumed those lost to follow up to be dead** | | | | |
| Male | Ref. | 1.13 (1.03, 1.24) | 1.14 (0.98, 1.34) | 1.59 (1.36, 1.86) |
| Female | Ref. | 1.02 (0.96, 1.10) | 1.40 (1.28, 1.55) | 1.73 (1.56, 1.93) |
| Total | Ref. | 1.05 (1.00, 1.12) | 1.31 (1.21, 1.42) | 1.68 (1.53, 1.83) |

† For total population, models were adjusted for demographics (age, sex, education, ethnicity, residence, marital status, occupation, and co-residence), smoking, drinking, physical activity, diet (fruit, vegetable, meat, fish, egg, bean, salted vegetable, sugar, tea, and garlic), leisure activities (housework, fieldwork, garden work, reading newspaper, raising pets, playing mahjong/cards, and watching TV or listening to videos), disease status, disability and year of the interview. For male and female, models were adjusted for all the above variables except for sex.

**Figure S1. The number of participants enrolled, lost, and died during the follow-up period by each survey year**

**1998**

**2000**

**2002**

**2005**

**2008**

4652

2530

998

332

Lost 837

Died 3285

Losti 561

Died 1561

Losti 264

Died 1268

Losti 203

Died 463

3599

1548

583

Losti 933

Died 1701

Losti 428

Died 1623

Losti 285

Died 680

**2011**

**2014**

117

42

Losti 47

Died 168

Losti 5

Died 70

229

92

Losti 76

Died 278

Lost i13

Died 124

1824

645

Losti 586

Died 2351

Losti 312

Died 867

277

137

Losti 85

Died 283

Losti 13

Died 127

1927

Losti 1019

Died 2563

683

291

Lostii302

Died 942

Lost i39

Died 353

2967

1416

Losti 1381

Died 3277

Losti 228

Died 1323

Enrollment in each survey year

Remaining sample size

**Figure S2. Unweighted multivariable-adjusted hazard ratios and 95% confidence intervals of all-cause mortality by cognitive function among Chinese aged ≥80 years**

Model 1: adjusted for continuous age and sex.

Model 2: adjusted for education, ethnicity, residence, marital status, and year of the interview based on model 1.

Model 3: adjusted for smoking, drinking, physical activity and diet (vegetable, egg, and garlic) based on model 2.

Model 4: adjusted for leisure activities (housework, field work, garden work, reading newspaper, raising pets, playing mahjong/cards, and watching TV or listening to videos) based on model 3.

Model 5: adjusted for disease status and ADL disability based on model 4.
